# Can urban e-commerce transformation improve economic resilience? a quasi-natural experiment from China

**DOI:** 10.1371/journal.pone.0304014

**Published:** 2024-05-31

**Authors:** Xiekui Zhang, Tongsheng Tang, Erhang Mo

**Affiliations:** 1 School of Economics & China-ASEAN Institute of Financial Cooperation, Guangxi University, Nanning, China; 2 School of Business Administration, South China University of Technology, Guangzhou, China; 3 The Research Base for Humanity Spirit and Social Development of Revolutionary areas in Guizhou, Yunnan, Guangxi and Their Border Areas, Baise, Guangxi, China; Xian Jiaotong University: Xi’an Jiaotong University, CHINA

## Abstract

The impact of urban e-commerce transformation on economic resilience can help a country improve its ability to resist risks and seize the initiative in economic development. This study examines the impact of the construction of the National E-commerce Demonstration City (NEDC) on economic resilience using the staggered different-in-differences approach using a sample of 282 Chinese cities from 2006 to 2020. The results show NEDC construction significantly strengthens urban economic resilience. This result remains robust after undergoing placebo test, exclusion of other policies interference, and examining endogeneity. Furthermore, noteworthy heterogeneity exists in the effect of NEDC construction on urban economic resilience, particularly in eastern, developed regions, and cities with high Internet penetration. The mechanisms analysis indicates that NEDC construction enhances urban economic resilience by expanding the scale of urban employment and enhancing market dynamism. Overall, this study refines the causal relationship between e-commerce development and urban economic resilience, providing empirical evidence and policy insights for China and other countries to enhance urban economic resilience and stabilize macroeconomic fluctuations.

## 1 Introduction

The 2008 global financial crisis and COVID-19 in 2020 have created many risks and challenges to the economic development of all countries, resulting in increased economic uncertainty globally [1]. As such, in today’s interconnected international economic landscape, economic resilience has attracted widespread attention among countries [2]. While regional economics and economic geography scholars have examined it, policymakers have also considered it as a regional development objective [3,4]. Meanwhile, with digital technology advancements, e-commerce has gained traction in the digital economy, gradually becoming a new engine of growth [5]. Its vigorous development has rapidly opened up the links of production, consumption, circulation and distribution between regions, and has played a positive role in stimulating economic vitality [6] and supporting economic growth [7]. As China is the world’s largest developing country [8], e-commerce development has profoundly affected all areas of the country’s economy and society. Especially since the outbreak of the COVID-19 pandemic, as the only major economy in the world which grew, China owes much of its economic momentum to the pivotal role played by e-commerce [7]. According to the National Bureau of Statistics (NBS), China’s annual e-transaction volume in 2022 has reached 43,829.9 billion yuan, or 36.21% of GDP. Thus, the development of e-commerce can enhance the Chinese economy’s resilience and strengthen its capacity to withstand shocks and recover.

The existing development achievements of e-commerce in China are closely related to the construction of the National E-commerce Demonstration City (NEDC) [9]. In 2009, Shenzhen became the pioneering NEDC, setting the groundwork for national e-commerce development. To further promote urban e-commerce transformation, the Chinese government issued the Guiding Opinions on the Creation of National E-commerce Demonstration Cities (hereafter, the Opinions) in 2011. The Opinions delineate the primary tasks of NEDC, aiming to establish it as a new initiative to promote economic transformation and cultivate new driving forces for economic growth, as well as resolve the main contradictions in economic development and seize the initiative for development [10]. NEDC can help to promote rapid e-commerce development, strengthen the resilience and transformative power of regional economies, and plays a crucial role in stabilizing economic fluctuations. However, given the vast expanse of China and diverse urban development systems, has the construction of NEDCs significantly enhanced China’s urban economic resilience? Is there regional heterogeneity in its impact? What are the pathways? Clarifying these issues will help us better understand the relationship between e-commerce development and economic resilience, and provide practical policy insights for stabilizing China’s macroeconomic fluctuations and achieving high-quality economic development.

Extant research has investigated the relationship between the construction of NEDC and social development, with most studies focusing on environmental pollution [11,12], carbon reduction [10,13], regional innovation [14,15], and economic development [16,17]. Only two articles have examined the economic effects of constructing NEDCs, with no study discussing how this construction affects economic resilience. Addressing these gaps, this study uses the staggered difference-in-differences (DID) method to explore the causal relationship between the construction of NEDC and economic resilience by analyzing data on 282 Chinese cities from 2006 to 2020.

This study contributes to the field in two key aspects. First, from a research perspective, we are the first to assess the impact of the construction of NEDC on economic resilience through the lens of e-commerce. We reveal the value of NEDC in terms of its economic effects and bridge the gap in understanding its relationship with urban economic fluctuations. Second, in terms of research methodology and content, this study constructs traditional and digital economic sector models by drawing on Feder’s design of export and non-export sector models [18]. We then analyze the direct impact of the construction of NEDC on urban economic resilience. Utilizing the staggered DID approach, we delve into the characteristics and pathways through which the construction of NEDC influences urban economic resilience. This provides valuable insights for Chinese policymakers and those from other countries to stabilize macroeconomic fluctuations, cultivate e-commerce industries, and enhance urban economic resilience.

The remaining article is structured as follows: Section 2 reviews the literature. Section 3 outlines the theoretical mechanisms and hypotheses. Section 4 introduces identification strategy and data acquisition. Section 5 reports and discusses the baseline empirical findings and robustness tests. Finally, we present the conclusions in section 6.

## 2 Literature review

This research is closely related to studies focusing on the definition and development effect of e-commerce, policy evaluation of the establishment of NEDC, defining economic resilience, and the measurement and influencing factors of economic resilience.

The first stream of literature focuses on defining e-commerce and evaluating its effectiveness. E-commerce can be considered as a commercial activity which utilizes information technologies to realize the buying, selling, and trading of commodities. It has diverse forms, such as online shopping, digital payment, logistics and transport, and product delivery [19,20]. Meanwhile, studies on e-commerce’s effectiveness focus on its impact on two aspects: economic development and environmental protection. First, in terms of its contribution to economic development, e-commerce is one of the fastest-growing industries in the digital economy with the greatest development potential [21]. Its emergence has promotes the integration of the real and digital economies [22]. Furthermore, its rapid growth can support the country’s economic development [23], promote financial market stability [24], contribute to rural revitalization and narrowing the urban-rural gap [25], and effectively alleviate the inequality of household consumption [26]. Second, in terms of environmental protection, some studies show that the impact of e-commerce development on the environment is a double-edged sword. On the one hand, e-commerce development can effectively reduce sulfur dioxide emissions [27], promote carbon emission reduction [28], and curb air pollution [29]. On the other hand, the expansion of urban e-commerce activities can further increase waste pollution [29] and cause environmental degradation [30].

The second research stream focuses on assessing the effects of the construction of NEDC, especially on its environmental and economic effects. Regarding the environmental effects, NEDC is a new urban development model for the Chinese government to actively improve air quality and reduce pollutant emissions. It can significantly reduce sulfur dioxide emissions, PM_2.5_ particulate matter concentration [11], carbon dioxide emissions [31], the use of agricultural fertilizers, pesticides, and agricultural films [12]; and improve energy utilization, energy conservation, environmental protection [32], and urban ecological environment [10]. Meanwhile, regarding the economic effects, NEDC has injected new vitality into China’s economic development and technological innovation. It accelerates the spatial agglomeration of service industries [33], enhances urban total factor productivity [16], promotes the development of a green economy [17], and facilitates regional industrial structure upgrading [31], enterprise digital transformation [34] and economic agglomeration [13].

The third research stream focuses on the definition of factors affecting economic resilience. First, the concept of resilience was originally proposed by Holling in 1973, and introduced into the ecological research framework for analyzing the ability of ecosystems to be maintained and repaired after being subjected to natural and anthropogenic shocks [35,36]. Subsequently, scholars have explored the concept of resilience in different fields and using perspectives, including in engineering and economics. Thus, there are diverse definitions of resilience, and a uniform definition is lacking [37]. In economics, Reggiani et al. were the first to introduce the concept of resilience into the study of spatial economic systems [38], which sparked widespread attention and related discussions among economists [39]. Martin defined economic resilience as the ability of economic entities to withstand crises, recover from them, self-renew, and reposition themselves through the study and analysis of possible responses of regional economies to recessionary shocks [40]. The author emphasized that economic resilience should encompass four dimensions: resistance, recovery, organization, and renewal. Next, Martin and Sunley defined macroeconomic resilience, arguing that an economic resilience is an economic system’s ability to withstand and recover from external shocks, and that it is the concentration of an economic system’s ability to effectively address economic risk challenges and reshape its development path [41]. The definitions of economic resilience by Martin and Martin and Sunley are widely recognized as they are consistent with an economic system characterized by disequilibrium, evolving dynamics, and complex adaptation. This study also adopts this definition.

The fourth research stream focuses on the measurement and influencing factors of economic resilience. First, studies have adopted two broad approaches for measuring economic resilience. One is the single core indicator approach, which gauges the persistence, resistance to contraction, and sensitivity of economic growth using metrics such as GDP growth or unemployment rates [42]. The other is the indicator system method, which systematically captures the evolving characteristics of economic resilience using various variables highly correlated with economic fluctuation [43]. Second, studies have shown that factors such as financial development [44], population agglomeration [45], regional innovation [46], industrial structure [47], government intervention [48], and digital transformation [49] can affect urban economic resilience.

In summary, several studies have assessed the effects of the construction of NEDC, and developmental trajectory and influencing factors of economic resilience. However, extant research mainly focuses on green economic efficiency and green total factor productivity. To our knowledge, no study has yet focused on the relationship between NEDC construction and economic resilience.

Thus, our study makes contributions from two aspects: First, from a research perspective, we are the first to consider the impact of e-commerce development on economic resilience. Exploiting the construction of NEDC as a quasi-natural experiment, we identify its influence on urban economic fluctuations and the underlying mechanisms, thus providing evidence on the relationship between e-commerce development and economic resilience. Second, from the perspective of research methods and content, we broaden the export and non-export dual-sector model developed by Feder [18]. By constructing a dual-sector model encompassing both traditional and digital economies, we integrate the construction of NEDC and economic resilience into a unified analytical framework. Utilizing this model, we identify the direct effects of NEDC on economic resilience. Furthermore, we employ the staggered DID way to verify the characteristics and pathways of NEDC’s impact on urban economic resilience.

## 3 Theoretical mechanism and hypotheses

The construction of NEDC is a significant catalyst propelling the growth of digital industries and digital technology advancements, as highlighted by Liu and Qiu and Wang et al. [10,13]. It can affect urban economic resilience both directly and indirectly. We draw inspiration from the framework proposed by Feder to establish a dual-sector model comprising both traditional and digital economic sectors [18]. Methodologically, we refer to the validation approaches used by Siddiqi et al., Zahid et al., and Ali et al. [50–52]. Through this, we investigate the direct impact of the construction of NEDC on urban economic resilience. Total output is defined as follows:

Y=T+D (1)

*T* and *D* represent the output of the traditional and digital economic sectors, respectively. Then, the production function for the digital sector of the economy can be defined as follows:

$$D=D(K_{D},L_{D})$$ (2)

Given that digital economy sectors, such as those in the NEDC, impact traditional sectors through digital technology, information networks, etc. Thus, the production function of the traditional sectors can be expressed as follows:

$$T=T(K_{T},L_{T},D)$$ (3)

*K* and *L* denote inputs of capital and labor factors to different sectors, respectively. Taking the time derivative of Eqs (2) and (3) yields the following relation [53]:

$$\frac{dD}{dt}=\frac{\partial D}{\partial K_{D}}\frac{dK_{D}}{dt}+\frac{\partial D}{\partial L_{D}}\frac{dL_{D}}{dt}$$ (4)

$$\frac{dT}{dt}=\frac{\partial T}{\partial K_{T}}\frac{dK_{T}}{dt}+\frac{\partial T}{\partial L_{T}}\frac{dL_{T}}{dt}+\frac{\partial T}{\partial D}\frac{dD}{dt}$$ (5)

$\frac{\partial D}{\partial K_{D}}、\frac{\partial D}{\partial L_{D}}、\frac{\partial T}{\partial K_{T}}、\frac{\partial T}{\partial L_{T}}$ and $\frac{\partial T}{\partial D}$ represent the marginal production of the two sectors with respect to capital and labor, and the marginal production of the traditional sector with respect to the digital sector, respectively. Since the digital and e-commerce production activities brought by the digital economy sector are inherently more innovative and productive than the traditional sector. Thus, we get the following relationship:

$$\frac{\partial D}{\partial K_{D}}/\frac{\partial T}{\partial K_{T}}=1+\lambda ,(\lambda >0)$$ (6)

$$\frac{\partial D}{\partial L_{D}}/\frac{\partial T}{\partial L_{T}}=1+\mu ,(\mu >0)$$ (7)

Now, taking the derivative of time with respect to Eq (1) yields the following:

$$\frac{dY}{dt}=\frac{\partial T}{\partial K_{T}}\frac{dK_{T}}{dt}+\frac{\partial T}{\partial L_{T}}\frac{dL_{T}}{dt}+\frac{\partial T}{\partial D}\frac{dD}{dt}+\frac{\partial D}{\partial K_{D}}\frac{dK_{D}}{dt}+\frac{\partial D}{\partial L_{D}}\frac{dL_{D}}{dt}$$ (8)

To simplify the analysis, assume *λ* = *μ* and substitute Eqs (4)–(7) into Eq (8). We introduced the design of Tian and Guo to obtain [42]:

$$\frac{dY}{dt}=\frac{\partial T}{\partial K_{T}}K+\frac{\partial T}{\partial L_{T}}\dot{L}+(\frac{\lambda }{1+\lambda }+\frac{\partial T}{\partial D})\frac{dD}{dt}$$ (9)

Further dividing by *Y*, the final collation gives:

$$\frac{\dot{Y}}{Y}=\frac{\partial T}{\partial K_{T}}\frac{K}{Y}+\frac{\partial T}{\partial L_{T}}\dot{\frac{L}{Y}}+(\frac{\lambda }{1+\lambda }+\frac{\partial T}{\partial D})\frac{\dot{D}}{D}\frac{D}{Y}$$ (10)

Clearly, the digital economy sector can contribute to increasing regional output. As suggested by Tian and Guo, a consistent rise in GDP is a crucial factor for economic systems to withstand external shocks, often reflecting the strength of regional economic resilience [42]. Therefore, we posit our first hypothesis as follows:

Hypothesis 1: The construction of NEDC can significantly enhance urban economic resilience.

Regarding indirect effects, e-commerce is a comprehensive industry strongly dependent on related services. The construction of NEDC promotes the further extension of e-commerce-related industrial chains, stimulates the spontaneous agglomeration of production services such as financial technology, warehousing, and logistics [33], and attracts more enterprises to relocate to the local area. This helps to expand the market size and facilitates employment growth [54,55]. Therefore, the construction of NEDC may optimize urban economic resilience through two channels: expanding the employment scale and enhancing market vitality.

On the one hand, the central government is encouraging pilot cities to leverage the e-commerce system for the further development of traditional commerce and service industries, guiding the entry of related sectors and promoting the deep integration of upstream and downstream e-commerce industry chains. Regional industrial cooperation is conducive to deepening the division of labor, alleviating labor mismatch, and reducing transaction costs, thereby further improving the quality of labor matching, and broadening the employment scale and employment opportunities for local workers [56,57]. Recent evidence suggests that employment can stabilize economic fluctuations. Digital development can bolster economic resilience by increasing the labor employment rate [58].

On the other hand, the evolution of e-commerce has facilitated the adoption of digital technology and the Internet, reducing the time and cost of information transfer. The openness and permeability of e-commerce enhance market participants’ access to information, alleviate information asymmetry among market players, improve the ability of transacting parties to reflect market changes, and play a positive role in expanding the market scale [44]. Steger pointed out that the expansion of consumer demand and market size stimulates economic vitality and growth [59]. Wang et al. echo the thoughts of Steger, finding that the construction of NEDC effectively inhibits market segmentation by invigorating market dynamics, promoting the free movement of commodities and diverse elements, and advancing the establishment and improvement of a unified national market [60]. This initiative has contributed to the reorganization and renewal of the urban economic system. Therefore, we formulate our second hypothesis as follows:

Hypothesis 2: The construction of NEDC fosters urban economic resilience by achieving employment stability and expanding market vitality.

## 4 Methodology

### 4.1 Method specification

To expedite e-commerce progress, and bolster the growth and strengthening of digital industries and technologies, the Chinese government has launched the construction of NEDC. In September 2009, the National Development and Reform Commission (NDRC) and the Ministry of Commerce identified Shenzhen as the inaugural NEDC. Afterwards, the NDRC, in conjunction with various government departments, announced the second, third, and fourth batches of NEDC in 2011, 2014, and 2017, respectively. Considering the conducive environment for quasi-natural experiments provided by the progressive reforms in China, we adopt the design proposed by Bertrand et al. to formulate the following model [61]:

$${RES}_{it}=\alpha_{1}+\beta_{1}{NEDC}_{it}+\theta_{1}X_{it}+\mu_{i}+\delta_{t}+\varepsilon_{it}$$ (11)

Where *RES*_*it*_ denotes urban economic resilience. We draw on Martin and employ China’s real GDP growth rates at the national and city levels to calculate urban economic resilience as shown in Eq (12) [40]. *NEDC*_*it*_ shows whether the city is an NEDC; it equals 1 if the city is classified as an NEDC in the current or future years, and 0 otherwise. *X*_*it*_ stands for a set of control variables. Based on Sun et al. [62] and Du et al. [44], and the above review, the selected control variables include the ratio of the tertiary industry to the secondary industry in GDP (indus), logarithm of number of patents granted in the current year in the city (lnA), ratio of urban fiscal expenditure to GDP (*gov*), logarithm of the total deposits and loans per capita of urban financial institutions (lnfinmar), logarithm of regional resident population divided by administrative units (lnpop), and logarithm of the total amount of postal services per capita (lninfo). *μ*_*i*_ and *δ*_*t*_ indicate the city and year fixed effects, respectively. Finally, *ε*_*it*_ represents a random perturbation term.


$${RES}_{it}=\frac{{(Y}_{it}-Y_{it-1})/Y_{it-1}-{(Y}_{nt}-Y_{nt-1})/Y_{nt-1}}{\left|{(Y}_{nt}-Y_{nt-1})/Y_{nt-1}\right|}$$ (12)

Where *Y*_*it*_ denotes the real GDP of the city *i* in period *t*, and *Y*_*nt*_ denotes the real GDP of the whole country in period *t*. If the value of *RES*_*it*_ is positive, it signifies a superior economic performance of city *i* in year *t* compared to the national average; and vice versa.

To further examine the role of the two channels of expanding the employment scale and improving market dynamics, we established the following mechanism model:

$${Mechnism}_{it}=\alpha_{2}+\beta_{2}{NEDC}_{it}+\theta_{2}X_{it}+\mu_{i}+\delta_{t}+\varepsilon_{it}$$ (13)

$${RES}_{it}=\alpha_{3}+\beta_{2}{NEDC}_{it}+\gamma_{3}{Mechnism}_{it}+\theta_{3}X_{it}+\mu_{i}+\delta_{t}+\varepsilon_{it}$$ (14)

Where *Mechnism*_*it*_ denotes the urban employment scale and market potential, and the other variables have the same meanings. Urban employment scale is proxied by the size of the labor force, which is measured as the logarithm of the average number of employees with a job (lnworker). Recognizing that nighttime lighting can provide insights into the market potential of a region, we draw on Wu et al. and utilizes the average nighttime light brightness *(*Nightlight) to characterize market potential [63].

### 4.2 Data sources

The study sample covers 282 Chinese cities from 2006 to 2020. Our primary data sources include the annual China City Statistical Yearbooks, EPS Global Statistics Platform, and Statistical Bulletin on national economic and social development of each region. We obtain the required patent data from the Chinese Research Data Services database. The list of NEDCs is retrieved from the NDRC’s official website. For some missing data, interpolation methods are employed. Due to missing data from some pilot cities, our research sample only covers 68 treatment group cities. Before testing the model, we performed covariance diagnostics. The maximum variance inflation factor (VIF) value is 4.09 and there is no multicollinearity problem.

Table 1 presents the descriptive statistics for all variables. The explanatory variable RES has a minimum value of -9.775 and a maximum value of 2.408, with a mean of 0.190. This indicates significant variations in the economic resilience across cities. Among explanatory variables, the mean value of 0.115 for NEDC indicates that a small sample of cities became pilot cities. Among the control variables, the distribution of lnfinmar is relatively flat, and indus, lnA, gov, lnpop, and lninfo vary significantly between cities by looking at the relationship between the mean, standard error, and maximum and minimum values. These variables are important factors influencing economic resilience. Finally, regarding the mechanism variable, the standard error value of Nightlight is as high as 8.842 compared to lnworker, indicating that market dynamism varies considerably across cities.

**Table 1:** Description of variables.

Variables	N	Mean	SD	Min	Max
RES	4230	0.190	0.490	-9.775	2.408
NEDC	4230	0.115	0.320	0	1
indus	4230	0.952	0.537	0.094	5.348
lnfinmar	4230	11.18	0.923	8.612	14.00
gov	4230	0.184	0.102	0.043	1.485
lnpop	4230	5.727	0.965	1.623	9.086
lnA	4230	6.785	1.823	1.386	12.31
lninfo	4230	4.547	0.941	2.090	8.449
lnworker	4230	3.453	0.832	0.307	6.649
Nightlight	4230	7.157	8.842	0.098	59.118

## 5. Empirical results

### 5.1 Baseline results

Table 2 illustrates the results of the impact of NEDC on urban economic resilience. In column (1), the coefficient of NEDC is statistically significant at the 5 percent level, with an estimated coefficient of 0.090 when the control variables are not considered. The significance of the NEDC coefficient in column (2) remains consistent after the inclusion of each control variable, with the estimated coefficient reaching 0.094. Thus, the construction of NEDC is beneficial for urban economy resilience, thereby supporting Hypothesis 1. This may be because the construction of NEDC accelerates the development of urban e-commerce and related industrial chains, and promotes the deep integration of the digital and real economies. Thus, cities can steadily advance towards high-quality economic development and effectively recover from the economic damage brought about by uncertain shocks. Meanwhile, an overly dominant tertiary industry proportion might adversely affect economic resilience. Specifically, cities that are too dependent on a certain industry or have too homogeneous industrial structures find it difficult to disperse risks when they are hit by strong shocks; then, more losses can easily happen. Only a diversified industrial structure can exhibit more sustainable resilience, consistent with Chen and Wang and He et al. [47,48].

**Table 2:** Baseline result.

	(1)	(2)
	RES	RES
NEDC	0.090**	0.094**
	(0.041)	(0.040)
indus		-0.217***
		(0.047)
lnfinmar		0.241***
		(0.072)
gov		-0.354
		(0.227)
lnpop		0.518***
		(0.174)
lnA		0.106***
		(0.025)
lninfo		0.023
		(0.025)
Constant	0.180***	-6.030***
	(0.005)	(1.406)
Controls	No	Yes
City effect	Yes	Yes
Year effect	Yes	Yes
N	4230	4230
Adj R-squared	0.171	0.201

Note

***, **, and * denote 1%, 5%, and 10% significance level, respectively.

### 5.2. Robust test

#### 5.2.1 Parallel trend

**Fig 1:**
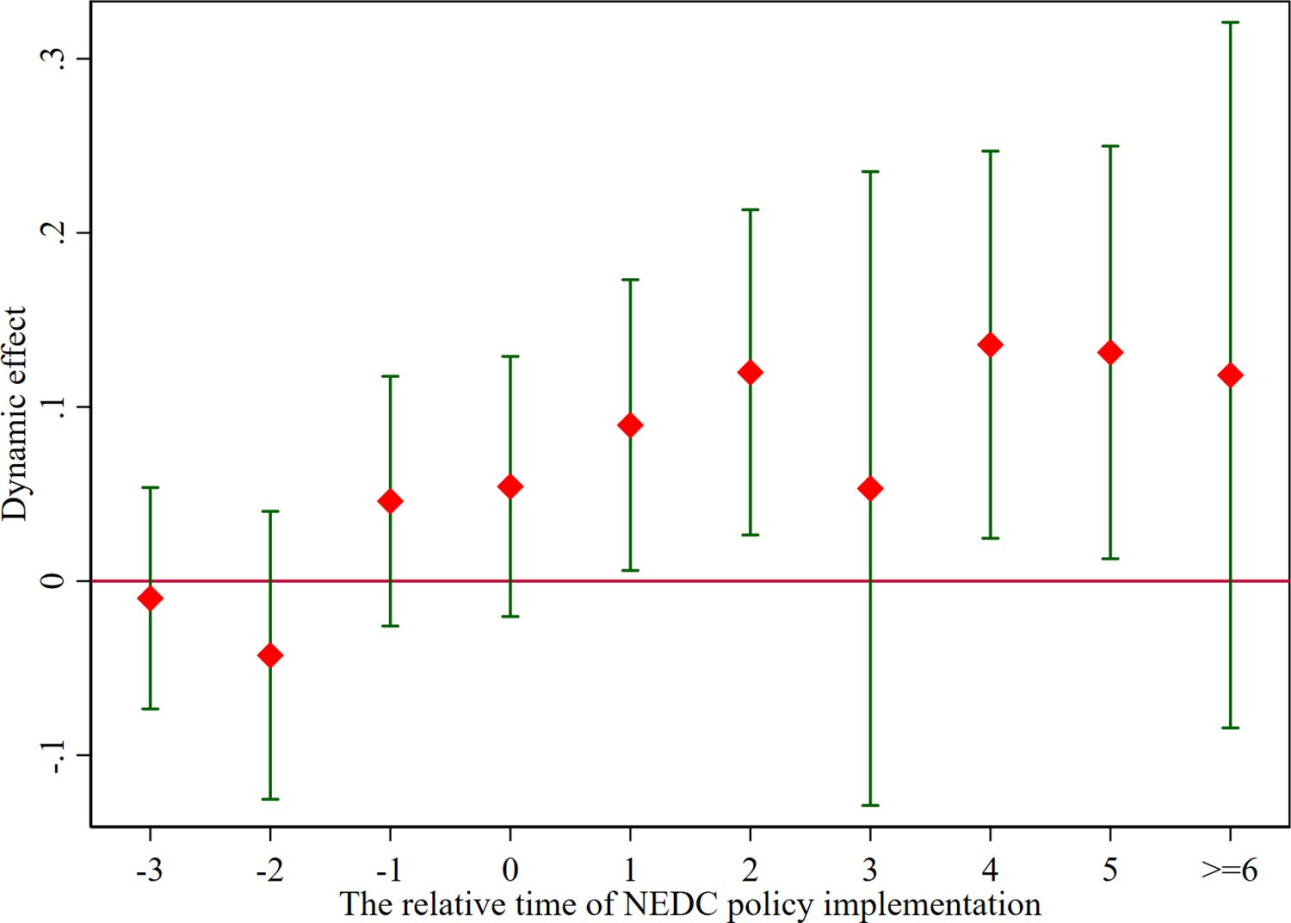
Parallel trend test.

The parallel trends assumption must hold to employ the DID approach. This entails verifying the absence of substantial trend changes in the resilience levels of pilot and non-pilot cities before the introduction of the NEDC. To identify such trends, we draw on the event study method proposed by Jacobson et al. and use the following regression [64]:

$${RES}_{it}=\alpha_{2}+\sum_{t=-3}^{6}\gamma_{t}D_{it}+\theta_{2}X_{it}+\mu_{i}+\delta_{t}+\varepsilon_{it}$$ (15)

where *RES*_*it*_ denotes urban economic resilience, *D*_*it*_ is a dummy variable for the current period and a city’s subsequent inclusion in the list pilot cities for the construction of NEDC, and *X*_*it*_ represents a series of control variables corresponding to Eq (1). We examine the period from four years before and six years after the policy implementation, with the fourth period preceding the policy serving as the baseline for parallel trend analysis. Fig 1 illustrates the dynamic influence of NEDC policy on urban economic resilience. The red solid points indicate the estimated coefficients, and the upper and lower vertical lines indicate the 95 percent confidence intervals. Clearly, the coefficient estimates of *γ*_*t*_ before NEDC policy implementation are insignificant, indicating a common pre-trend for pilot and non-pilot cities. After the implementation of the NEDC policy, the coefficient *γ*_*t*_ is significant and has a continuous dynamic effect.

#### 5.2.2 Placebo test

**Fig 2:**
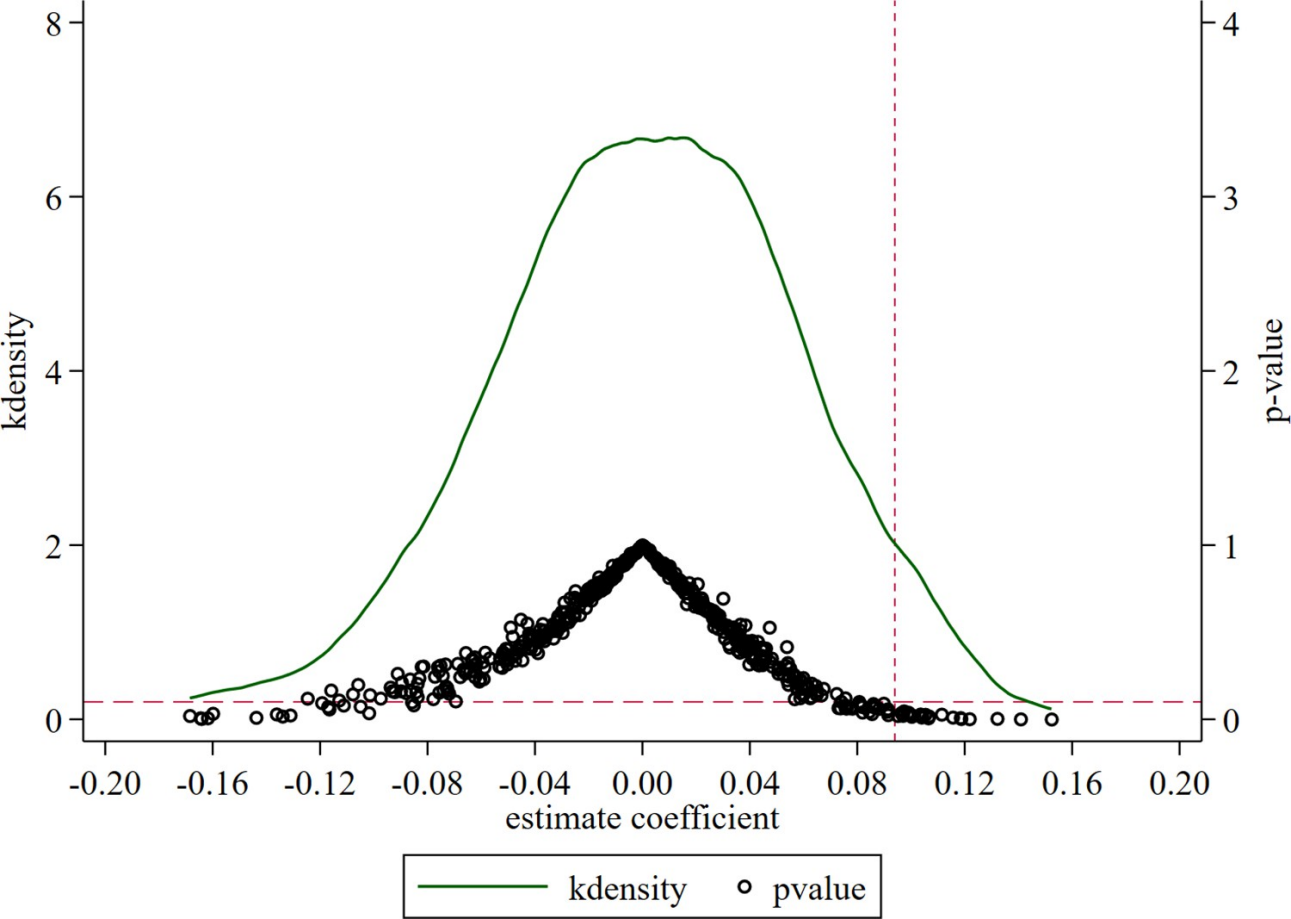
Placebo test.

Although we add control variables affecting economic resilience, and time and city fixed effects in the basic regression, there might persist unobserved factors that bias the benchmark regression. Therefore, adopting the treatment method used by Cai et al., we conduct a random simulation of 500 shocks. Each time, 68 cities were randomly selected from the sample as the treatment group and the rest were classified as the control group for a counterfactual test [65]. Finally, we obtain the estimated coefficient, corresponding p values, and kernel density distribution of the influence of the construction of pseudo-NEDC on urban economic resilience, as shown in Fig 2.

Compared with actual policy impact, the regression coefficients obtained by the pseudo-policy shock are mainly concentrated around the value of 0. Further, p values are mostly insignificant. This suggests that the benchmark regression results are free from unobservables and are robust.

#### 5.2.3 Eliminate other policies interference

China’s progressive reforms indicate that a policy implemented in certain cities is not unique. To accurately assess the "net effect" of the construction of NEDC on economic resilience, we account for policies potentially impacting economic resilience. First, we exclude the influence of the low-carbon transformation strategy. Liu found that China’s low-carbon city pilot policy (LCCP) effectively shields against external shocks and sustains economic stability [43]. Second, we remove the shock of information infrastructure construction on economic resilience. Zhang demonstrated that broadband reduced China’s economic losses during the pandemic and made China more resilient than other countries, which may be due to the implementation of the "Broadband China" plan (Broadband) [66]. Third, we eliminate the effect of innovation-driven policy. Some evidence suggests that the national innovative city pilot policy (NICP) has fostered green technology innovation, which is crucial for China to enhance its resilience to risks and achieve high-quality economic development [67,68]. We integrate the aforementioned policy shocks into the benchmark model. The findings presented in Table 3 indicate that the main effect remains robust even when considering the impact of other policies.

**Table 3:** Eliminate other policies’ interference.

	(1)	(2)	(3)	(4)
	RES	RES	RES	RES
NEDC	0.099**	0.098**	0.076*	0.084**
	(0.040)	(0.043)	(0.039)	(0.041)
Constant	-6.058***	-6.047***	-6.007***	-6.058***
	(1.410)	(1.402)	(1.404)	(1.403)
Controls	Yes	Yes	Yes	Yes
LCCP	Yes			Yes
Broadband		Yes		Yes
NICP			Yes	Yes
City effect	Yes	Yes	Yes	Yes
Year effect	Yes	Yes	Yes	Yes
N	4230	4230	4230	4230
Adj R-squared	0.201	0.200	0.201	0.201

Note

***, **, and * denote 1%, 5%, and 10% significance level, respectively.

#### 5.2.4 Endogeneity treatment

As NEDC was introduced in batches, its far-reaching economic and social impacts may have encouraged non-pilot cities to apply for the policy enthusiastically. This could lead to a reverse causality between the construction of NEDC and economic resilience. Therefore, we collected the number of post-stations in the city during the Ming Dynasty as an instrumental variable (IV) for the construction of NEDC. As an important gateway for information transit and transmission, the location of these post stations is correlated with the development of modern information infrastructures. Cities that have built post stations in history may have higher probability of becoming NEDCs by virtue of their advantages such as convenient transportation, developed logistics, and a developed economy. Meanwhile, the Ming Dynasty post stations themselves do not directly affect regional economic resilience. Since cross-section data cannot be directly applied to panel data, this study adopts the idea by Nunn and Qian and Zhou and Li, and interacts with this IV with e-commerce transaction volume to address the possible causal effect [14,69].

The results reported in Table 4 show that the more post stations a city has, the more likely that it will be named as a NEDC. The second stage results show that, despite the core variable coefficients being inflated by a factor of about ten times compared to the resulting coefficients in the fundamental regression, one can argue that the construction of NEDC significantly contributes to urban economic resilience.

**Table 4:** Instrumental variable outcomes.

	(1)	(2)	(3)
	NEDC	RES	RES
IV: Ming Dynasty post station×E-commerce	0.000***		
	(0.000)		
NEDC		1.669***	1.366**
		(0.587)	(0.618)
Cragg-Donald Wald F statistic		19.522	16.391
		(16.38)	(16.38)
Controls	Yes	No	Yes
City effect	Yes	Yes	Yes
Year effect	Yes	Yes	Yes
N	4230	4230	4230

Note

***, **, and * denote 1%, 5%, and 10% significance level, respectively.

#### 5.2.5 Other robustness tests

We performed the following four additional tests: (1) winsorized all continuous variables by 1 percent to avoid the interference of outliers; (2) designed a basket of variables as a proxy supplement for economic resilience, as shown in Table 5; (3) utilized the propensity score matching-DID method for the one-to-two nearest neighbor matching to address the selectivity bias of the construction of NEDC; and (4) as the sample cities were hit by the COVID-19 pandemic in 2020, 2020 was designated as 1 and the other years as 0 in the benchmark regression to isolate the pandemic’s effects. Unfortunately, global financial crisis only affected specific cities, making it challenging to quantify its effects. Note that the Herfindahl-Hirschman Index is calculated based on the sum of the squares of the share of industry *i* in GDP (*i* = 1,2,3). This index serves as a measure of the degree of diversification in a region’s industries. A higher index value indicates lower diversity in the region’s industries, signaling weaker resistance to risk; therefore, the index is negatively attributed. The methodology for calculating the advanced industrial structure is drawn from Liu [43].

**Table 5:** Evaluation indicators of urban economic resilience.

Category	Sub-indicators	Interpretation of indicators	characteristic
Resistance & Persistence	Scale of economy	GDP per capita	+
	Risk resistance of residents	Resident savings balance	+
	Concentration of industries	HHI index	−
	Foreign trade dependence	Total import and export trade volume /GDP	−
Organization & moderation	Investment support	Logarithm of per capita fixed asset investment	+
	Size of market	Total retail sales of consumer goods /GDP	+
	Degree of financial self-sufficiency	General budget revenue/general budget expenditure	+
Transformation & renewal	Driven by innovation	Science and technology expenditure	+
	Transformation of industries	Advanced industrial structure	+
	Rate of urbanization	Urban permanent population/permanent population	+
	Support of talents	Number of Internet users	+

Table 6 summarizes the outcomes of the aforementioned treatments and affirms that the construction of NEDC on the economic resilience of cities is positive and robust. We observe significant negative effects of COVID-19 on economic stability. This may be because most cities in China actively initiated emergency response measures to further prevent the spread of COVID-19, including implementing regional lockdowns and individual quarantines [1]. These initiatives have rapidly changed China’s economic operating environment, creating numerous challenges to China’s economic growth, such as slowing investment growth, weak increase in consumption, and low demand for exports, thus affecting the economic resilience of China’s cities [70].

**Table 6:** Various robustness treatments.

	(1)	(2)	(3)	(4)
	RES_w	RES-sub	PSM-DID	RES
NEDC	0.085**	0.017***	0.094**	0.094**
	(0.035)	(0.003)	(0.042)	(0.040)
Constant	-5.187***	-0.323***	-5.849***	-5.666***
	(1.185)	(0.160)	(1.417)	(1.349)
Covid				-0.729***
				(0.162)
Controls	Yes	Yes	Yes	Yes
City effect	Yes	Yes	Yes	Yes
Year effect	Yes	Yes	Yes	Yes
N	4230	4230	4167	4230
Adj R-squared	0.310	0.890	0.204	0.153

Note

***, **, and * denote 1%, 5%, and 10% significance level, respectively.

### 5.3 Mechanism analysis

Table 7 showcases the results for two potential channels we consider: employment scale and market potential. Column (1) reveals a substantial enhancement in urban employment levels due to NEDC, underscoring its pivotal role in augmenting employment. This may be because the construction of NEDC has enabled pilot cities to receive policy support, such as start-up subsidies and tax incentives, attracting a large number of e-commerce-related firms to relocate to the local area. This influx of businesses provided ample employment opportunities for local residents, resulting in a significant surge in regional employment [71]. Next, the coefficient value of lnworker in column (2) is 0.335 and significant at the 1% level. Thus, employment can significantly enhance economic resilience. A stable labor market helps stimulate local productive capacity, consumption levels, and economic development, accelerates the pace of urban economic recovery and adjustment, and thus, improves urban economic resilience [62].

**Table 7:** Mechanism checks.

	(1)	(2)	(3)	(4)
	lnworker	RES	Nightlight	RES
lnworker		0.335***		
		(0.091)		
NEDC	0.072***	0.070*	1.188***	0.081**
	(0.034)	(0.041)	(0.395)	(0.040)
Nightlight				0.011**
				(0.005)
Constant	-0.293	-5.932***	-11.237	-5.907***
	(0.904)	(1.353)	(13.256)	(1.362)
Controls	Yes	Yes	Yes	Yes
City effect	Yes	Yes	Yes	Yes
Year effect	Yes	Yes	Yes	Yes
N	4230	4230	4230	4230
Adj R-squared	0.961	0.213	0.958	0.202

Note

***, **, and * denote 1%, 5%, and 10% significance level, respectively.

Columns (3) and (4) delve into the mediating role of market vitality in the relationship between NEDC and urban economic resilience. We find that the construction of NEDC has effectively expanded the regional market scale. By promoting the quality and efficiency of the urban economy, it in turn has significantly elevated urban economic resilience. This may be because the construction of NEDC has improved the policy and business environments for e-commerce development in the pilot cities, removing the barriers in traditional markets. This not only reduces the cost and threshold of entrepreneurship, providing new opportunities for entrepreneurs to start businesses, but also promotes significant changes in business models, expands market boundaries, and enhances dynamism [72]. Enhancing market potential is crucial for promoting high-quality economic development in cities, thereby strengthening the organizational capacity and renewal capability of urban economic systems [60]. In summary, urban employment scale and market potential are two pathways through which the construction of NEDC positively influences urban economic resilience.

### 5.4 Heterogeneity analysis

**Fig 3:**
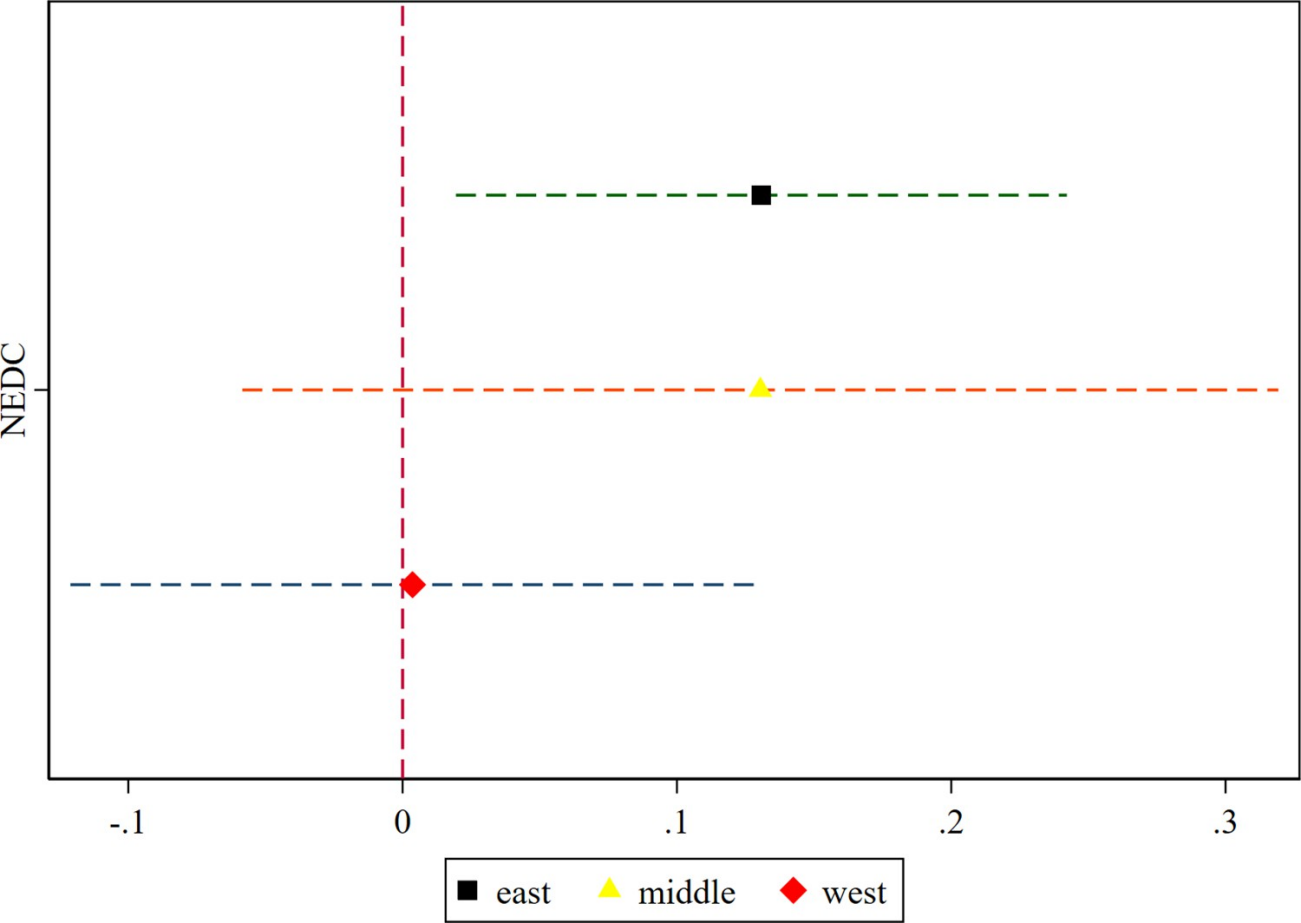
Heterogeneity by region.

**Fig 4:**
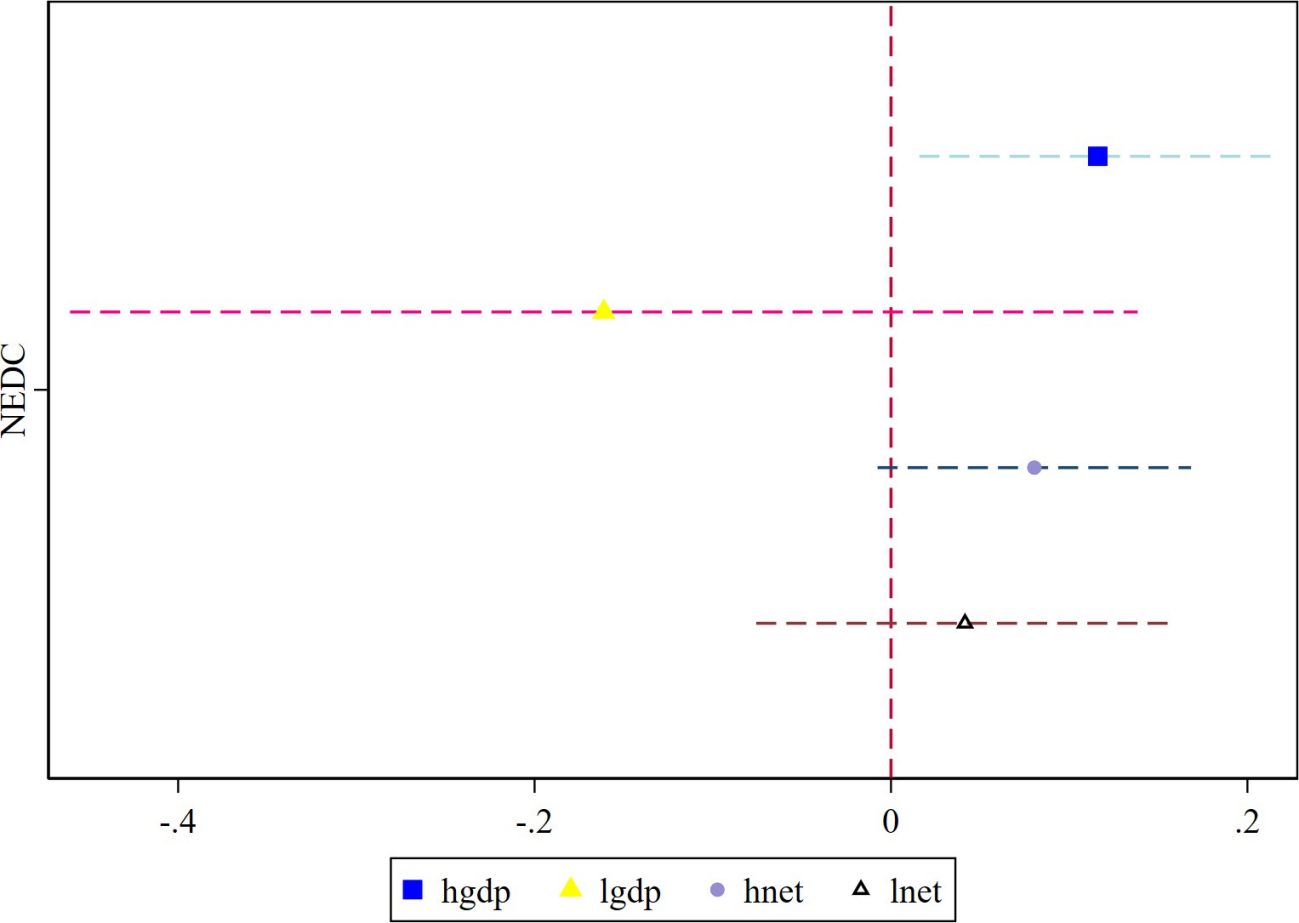
Heterogeneity by economic and internet development.

This study explores the heterogeneous effects of the construction of NEDC based on geographic location, economic development, and Internet development levels. Table 8 presents the results. Figs 3 and 4 reflect the coefficient changes and confidence intervals for NEDC in the heterogeneity analysis, where the estimated coefficients are represented by different colored shapes and dotted lines indicate the 10% confidence intervals.

**Table 8:** Heterogeneity analysis.

	(1)	(2)	(3)	(4)	(5)	(6)	(7)
	east	mid	west	hgdp	lgdp	hnet	lnet
NEDC	0.131**	0.130	0.004	0.116**	-0.161	0.080*	0.042
	(0.056)	(0.095)	(0.063)	(0.051)	(0.152)	(0.045)	(0.060)
Constant	-8.557**	-2.761	-6.195**	-2.323	-6.926***	-2.961	-6.088***
	(3.312)	(2.015)	(3.096)	(2.365)	(1.877)	(3.381)	(1.756)
Controls	Yes	Yes	Yes	Yes	Yes	Yes	Yes
City effects	Yes	Yes	Yes	Yes	Yes	Yes	Yes
Year effect	Yes	Yes	Yes	Yes	Yes	Yes	Yes
N	1500	1485	1245	2175	2030	1218	2989
Adj R-squared	0.299	0.189	0.207	0.208	0.385	0.188	0.289

Note

***, **, and * denote 1%, 5%, and 10% significance level, respectively.

First, the unbalanced and insufficient regional development in China may influence the effect of NEDC on urban economic resilience. We categorize China into eastern, central, and western regions, and investigate the influence of NEDC on economic resilience. Clearly, the construction of NEDC enhances resilience in the eastern region, while the positive coefficients in the central and western regions lack statistical significance. Cities in eastern China are at the forefront of the country’s efforts for reform and opening up. They are relatively advanced in economic development, not only possessing greater advantages in economic agglomeration and optimization of industrial structure but also being more mature in the development of the digital economy and information infrastructure. Since the construction of NEDC requires support from various factors such as urban human capital, digital industries, network infrastructure, and policy incentives, this may have led to a more pronounced promotional effect of NEDC on the eastern region’s economic resilience.

Second, to further examine the regional heterogeneity, we partition the sample into high and low-economic development groups based on the average GDP per capita. We find that the construction of NEDC serves as the "icing on the cake" for the timely economic recovery in developed regions. However, it fails to provide less-developed regions with fuel for recovery. Thus, the higher the level of economic development, the more advantageous it is for regions to adapt their economic structural patterns via e-commerce.

Third, the level of Internet development is intricately linked to the growth of regional e-commerce. The connectivity of urban networks serves as the foundation for regional e-commerce operations. We use the number of urban Internet users as a proxy variable for the level of regional Internet development, and categorize regions into high and low-Internet-level groups based on the mean value. We find that the impact of the construction of NEDC on urban economic resilience is more pronounced in regions with more robust Internet facilities. This is because the spread of broadband networks has facilitated the dissemination and exchange of information, increasing productivity in digital sectors, such as that of e-commerce platforms, thereby shifting production factors to these sectors. This has enhanced resource allocation efficiency. Further, the effective integration of resources has improved the organizational capacity of urban economic systems, ultimately driving growth in social output.

## 6 Conclusions

The construction of NEDC stands as an important initiative aimed at encouraging pilot cities to explore the healthy development of e-commerce, and their own enhance competitive advantage and resilience. Utilizing panel data encompassing 282 Chinese cities spanning from 2006 to 2020, this study focuses on the construction of NEDC to delve into the relationship between NEDC and urban economic resilience. We find that urban economic resilience notably increases due to the construction of NEDCs, increasing by 0.094 units in pilot cities compared with non-pilot cities. This result holds even after undergoing a battery of robustness tests. Furthermore, NEDC improves urban economic resilience through two channels: expanding the employment scale and promoting market vitality. Finally, the effect of the construction of NEDC on urban economic resilience is more pronounced in the eastern regions, economic developed regions, and cities with high Internet penetration. Some results are consistent with Lv and Chen, who indirectly found that NEDC construction can promote economic development [17]. However, compared to their use of carbon emissions, energy, and economic output to construct green economic efficiency as explanatory variables, we measure economic resilience from a resilience perspective using both the single-indicator and indicator system approaches. Thus, we emphasize the importance of economic fluctuations and ability of the economic system to resist risks. Furthermore, given that Lv and Chen categorized NEDC as part of digital transformation, Ji ang Huang found that the Broadband China strategy and digital transformation significantly increase the economy’s resilience to external shocks [49]. However, the authors failed to consider the driving role of the construction of NEDC. We fill this gap, revealing the promoting effects of e-commerce development and digital transformation on economic resilience.

Our insights have the following policy implications. First, the implementation of NEDC should be augmented to optimize the e-commerce industrial layout. Specifically, the positive and reinforcing role of NEDC should be leveraged to achieve stable employment and expand the market size, steer cities toward high-quality e-commerce-oriented development, and effectively address uncertainty shocks and volatility. On the one hand, based on their own economic structure and infrastructure conditions, the eastern region and high-Internet penetration cities should explore appropriate e-commerce development paths for the transformation of old and new industries. This is essential for achieving the convergence of traditional and modern dynamics, fostering distinct e-commerce applications, and service models. On the other hand, the central and western regions along with low-Internet penetration cities should strengthen their digital infrastructure, improve the urban business and policy environments, attract e-commerce platforms and enterprises, and leverage e-commerce in promoting resilience.

Second, stakeholders must work to establish a coordinated development mechanism for e-commerce and actively build a network of urban e-commerce. Here, the government’s role encompasses augmenting top-level e-commerce planning, providing financial support to e-commerce demonstration zones, encouraging platform cooperation, and addressing fundamental issues in e-commerce development. In particular, it should simplify the procedures of establishing companies, e-commerce logistics, and storage to facilitate the fusion of the digital and real economies. In addition, e-commerce enterprises should improve the application and service capabilities of e-commerce, strengthen technological innovation and platform association, build the agglomeration of urban e-commerce enterprises, optimize resource efficiency allocation in the industry, and provide the endogenous impetus for enhancing urban economic resilience.

This study has some limitations. First, we only focus on prefecture-level cities and analyze the impact of the construction of NEDC on urban economic resilience at the macro level. Future studies could focus on the micro level, and undertaking county- and enterprise-level analyses to further investigate the influence and pathways of NEDC on economic resilience at the micro level. Second, we only analyzed the overall impact of NEDC on urban economic resilience. Scholars should conduct in-depth analyses of the developmental trajectories and trends of NEDC’s influence on economic resilience across different cities. This can help in formulating more targeted interventions. Finally, the conclusions drawn here are based on an analysis of urban areas from China. As such, the empirical evidence and policy recommendations are primarily applicable to China, with limited generalizability elsewhere. The next step could involve extending our research framework and empirical methods to other developed, developing, and underdeveloped countries.

## Supporting information

S1 Data (XLSX)
